# Characterization data of reference cement CEM III/A 42.5N used for priority program DFG SPP 2005 “Opus Fluidum Futurum – Rheology of reactive, multiscale, multiphase construction materials”

**DOI:** 10.1016/j.dib.2020.105524

**Published:** 2020-04-12

**Authors:** Z.C. Lu, M. Haist, D. Ivanov, C. Jakob, D. Jansen, M. Schmid, P.A. Kißling, S. Leinitz, J. Link, V. Mechtcherine, J. Neubauer, J. Plank, W. Schmidt, C. Schilde, C. Schröfl, T. Sowoidnich, D. Stephan

**Affiliations:** a Department of Civil Engineering, Technische Universität Berlin, 13355 Berlin, Germany; b Since 02/2019: Institute of Building Materials, Leibniz Universität Hannover, 30167 Hannover, Germany; c Until 01/2019: Institute of Concrete Structures and Building Materials (IMB), Karlsruhe Institute of Technology, 76131 Karlsruhe, Germany; d Institute for Particle Technology, Technische Universität Braunschweig, 38106, Braunschweig, Germany; e GeoZentrum Nordbayern, Mineralogy, Friedrich-Alexander Universität Erlangen-Nürnberg, 91054 Erlangen, Germany; f Department of Chemistry, Technische Universität München, 85748 Garching, Germany; g Institute of Physical Chemistry and Electrochemistry, Leibniz Universität Hannover, 30167 Hannover, Germany; h Bundesanstalt für Materialforschung und - prüfung (BAM), 12205 Berlin, Germany; i Institute of Construction Materials, Technische Universität Dresden, 01159 Dresden, Germany; j F.A. Finger-Insitute for Building Materials, Bauhaus-Universität Weimar, 99421 Weimar, Germany

**Keywords:** Cement, Slag, Characterization, DFG SPP 2005

## Abstract

Two types of cements were selected as the reference cement in the priority program 2005 of the German Research Foundation (DFG SPP 2005). A thorough characterization of CEM I 42.5 R has been made in a recent publication [1]. In this paper, the characterization data of the other reference cement CEM III/A 42.5 N are presented from the aspects of chemical and mineralogical compositions as well as physical and chemical properties. The characterization data of the slag, which is the second main constituent of this specific cement besides the clinker, are presented independently. For all data received, the mean values and the corresponding errors were calculated. The data shall be used for the ongoing research within the priority program. Also, researchers from outside this priority program can benefit from these data if the same materials are used.

Specifications table

**Table:** 

Subject	Ceramics and Composites
Specific subject area	Building materials; Cement
Type of data	Table; Image; Graph; Figure
How data was acquired	XRD; SEM; EN 196–1: 2016; EN 196–2: 2013; EN 196–3: 2016; EN 196–6: 2018; EN 196–11: 2018; EN 1097–7: 2008; ISO 13,320: 2009; ISO 9277: 2010
Data format	Raw; Analyzed
Parameters for data collection	Chemical composition; Phase contents; Density; Specific surface area; Particle size; Calorimetry; Water demand; Setting time; Mechanical strength
Description of data collection	Firstly a thorough characterization of CEM III/A 42.5 N sample was made by in total 10 research groups. Afterwards, the data were collected and compared in this paper. Furthermore, the mean values and the corresponding errors were calculated based on the collective data.
Data source location	Seven universities, one research institute, and one company as shown in Table 1
Data accessibility	The data are included in this article

## Value of the data

• The data are useful because a thorough characterization of the CEM III/A 42.5 N sample was conducted, which is the basis for further research in the DFG SPP 2005 priority program.
• All research groups involved in the DFG SPP 2005 priority program can use these data for their research work and cite this paper instead of publishing the data several times in every individual paper. The researchers outside the priority program can also use these data if the same materials are used.
• The comparison between the future characterization date on this cement and the data shown here can be made to check the aging degree of this cement during storage.
• The data have a statistical significance because, in total, ten research groups from seven universities, one research institute and one company, were involved in collecting data on the CEM III/A 42.5 N sample using renown standard procedures.
• For all properties, the mean values and the corresponding errors were calculated based on the collected data from the test results.

## 1 Data

**Fig. 1:**
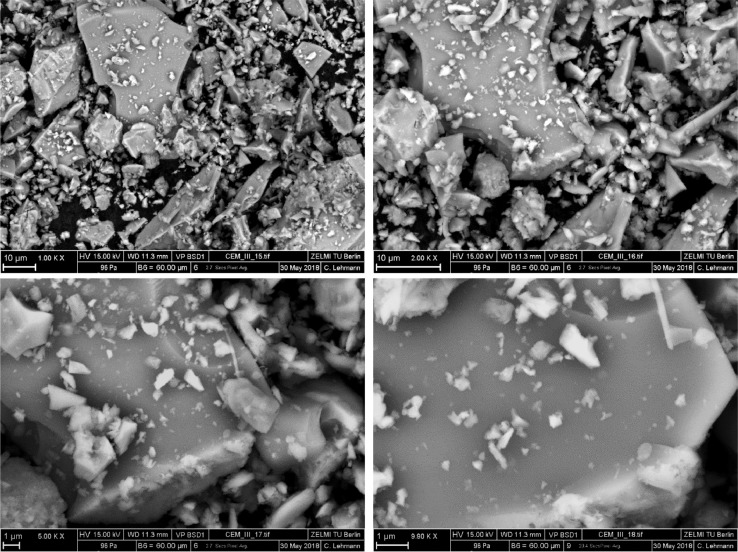
SEM pictures of the CEM III/A 42.5 N with different magnifications.

The affiliations and the corresponding abbreviations of the participants are listed in Table 1. The SEM pictures of CEM III/A 42.5 N sample with different magnifications are shown in Fig. 1.

**Table 1:** Universities, research institute and the company involved in the characterization.

Acronym	Affiliation
BAM	Bundesanstalt für Materialforschung und -prüfung
BUW	Bauhaus-Universität Weimar
FAU	Friedrich-Alexander Universität Erlangen-Nürnberg
Heidelberg	HeidelbergCement AG
LUH	Leibniz Universität Hannover
TUB	Technische Universität Berlin
TUBS	Technische Universität Braunschweig
TUDD	Technische Universität Dresden
TUM	Technische Universität München

### 1.1 Characterization data of the cement

#### 1.1.1 Oxide composition and phase contents

**Fig. 2:**
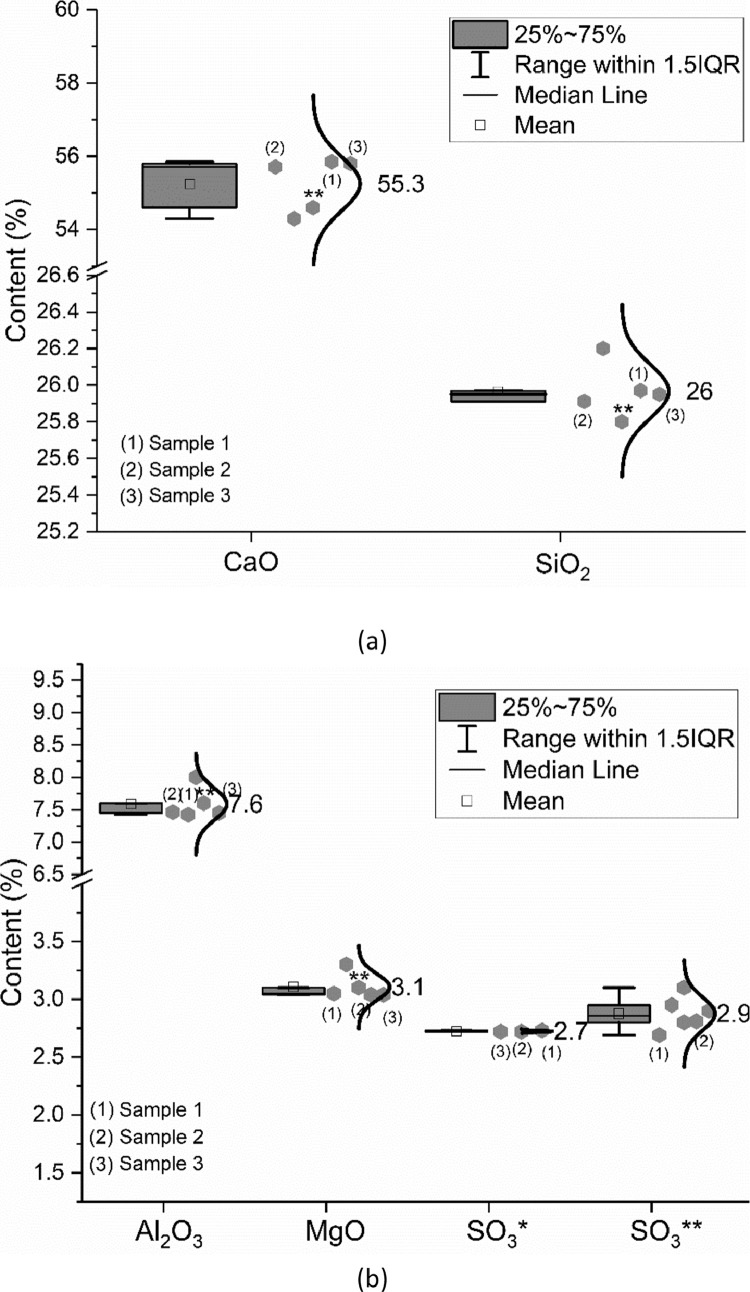

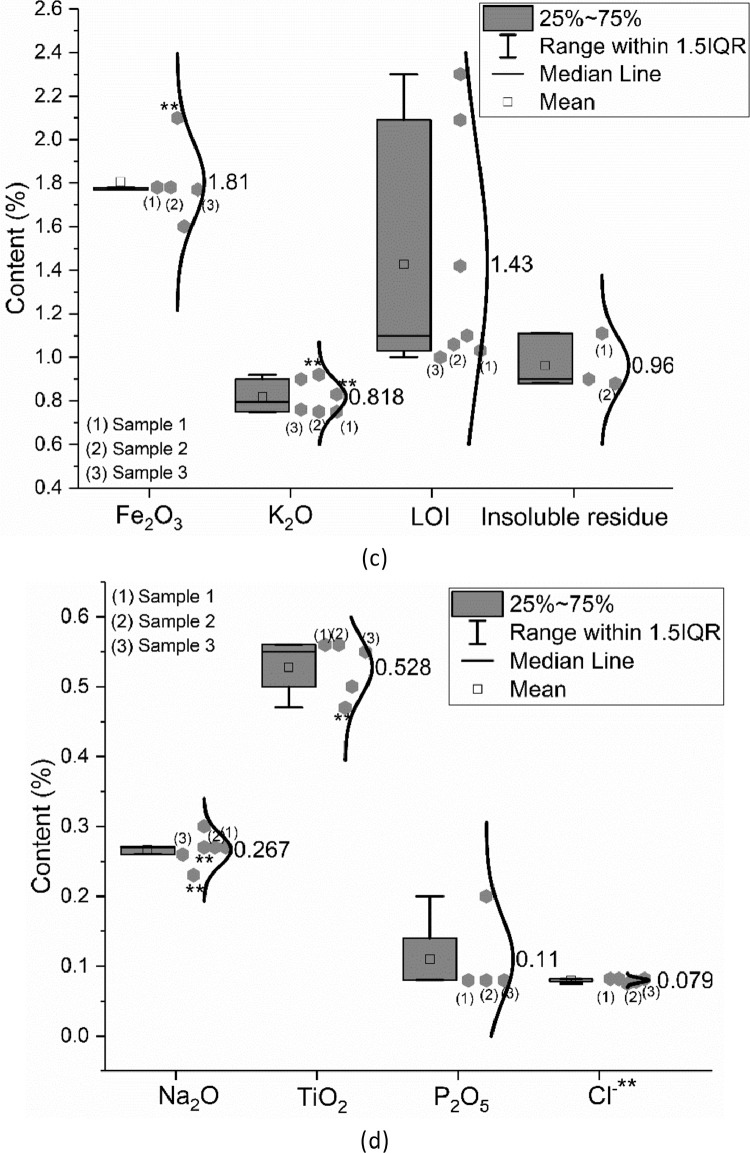
Oxide composition of CEM III/A 42.5 N; (a) CaO and SiO_2_; (b) Al_2_O_3_, MgO and SO_3_; (c) Fe_2_O_3_, K_2_O, loss on ignition (LOI) and insoluble residue; (d) Na_2_O, TiO_2_, P_2_O_5_ and Cl^−^.

The oxide composition (CaO, SiO_2_, Al_2_O_3_, Fe_2_O_3_, SO_3_, MgO, K_2_O, Na_2_O, TiO_2,_ and P_2_O_5_), insoluble residue as well as the loss on ignition (LOI) of the CEM III/A 42.5 N were measured following EN 196–2: 2013 [2]. Up to five groups were involved in the oxide composition characterization. The data are shown in Fig. 2. As described in [1], the data marked as (1) to (3) were provided by one research group. They measured the materials from one single batch but different bags. In Fig. 2(b), SO_3_* means the value obtained by X-ray fluorescence analysis (XRF) and SO_3_** indicates the value captured by the wet chemistry method. The same meaning of ** applies for any other data shown in Fig. 2. Unless otherwise stated, the oxide composition shown in Fig. 2 is measured based on XRF analysis.

The meanings of the legend in the figures are also clarified in [1]. IQR means the interquartile range, namely the range between 25th and 75th percentiles. The error bar shows the range within 1.5 times of the IQR. The median line indicates the 50th percentile. The mean value is calculated based on data from all samples within the 1.5 IQR range and does not include outliers.

Table 2 shows the phase contents of CEM III/A 42.5 N based on the data from two different groups (#1 and #2) through the method of powder-XRD combined with the quantification of the patterns according to the Rietveld refinement method [3]. Group #2 used additionally the external G-factor method [4] for an absolute quantification of all crystaline phases and the slag. For quantification of the slag the PONKCS method [5] was applied.

**Table 2:** Phase contents of CEM III/A 42.5 N measured by two different groups.

	Alite	Belite	Arcanite	C_3_A (orth)	C_3_A (cub)	C_4_AF	Anhydrite	Bassanite	Gypsum	Calcite	Quartz	Periclase	Slag	Sum
#1	28.34	10.49	–	1.73	4.44	–	1.73	–	–	3.29	0.60	0.40	45.36	99.80
#2	29.00	7.10	0.60	1.70	3.50	3.40	2.30	2.00	0.30	2.70	0.40	–	45.90	99.20

#### 1.1.2 Physical properties

**Fig. 3:**
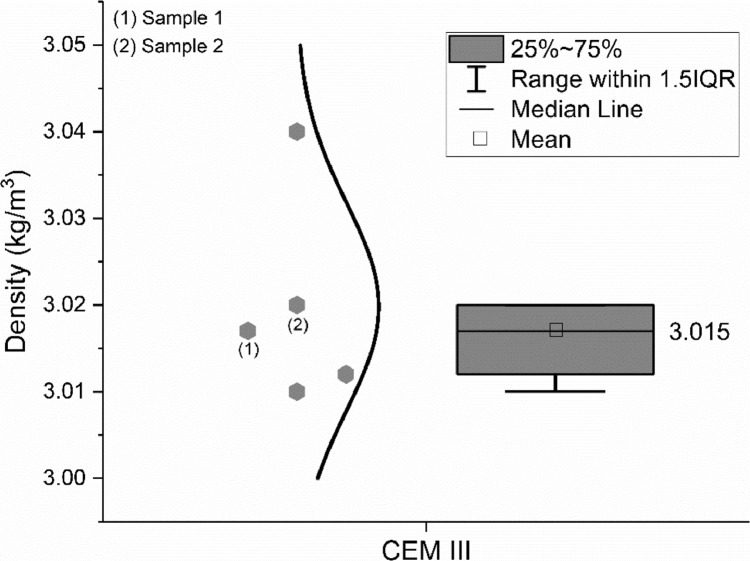
True density of the CEM III/A 42.5 N.

The true density of the CEM III/A 42.5 N was measured by Helium pycnometer method according to standard EN 1097–7: 2008 [6]. Four different groups were involved in the characterization and the data are shown in Fig. 3.

**Fig. 4:**
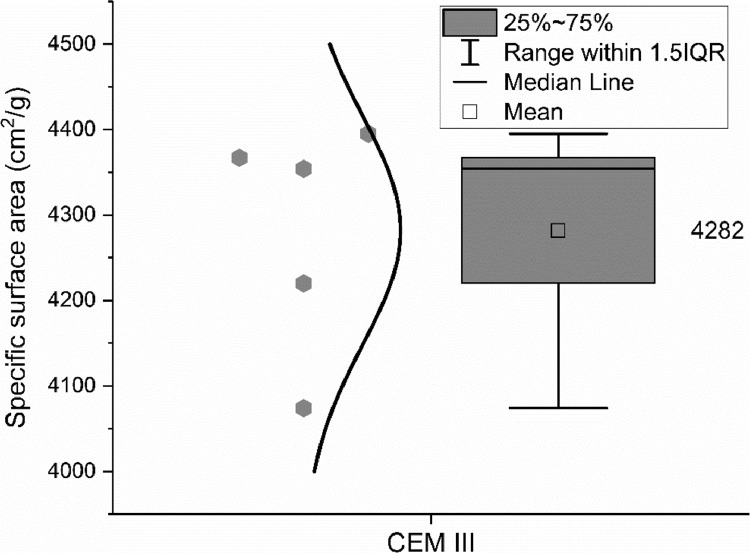
Specific surface area of the CEM III/A 42.5 N measured by the Blaine method.

The specific surface area of the CEM III/A 42.5 N was measured by the Blaine method, according to EN 196–6: 2018 [7]. Five different groups measured the particle size by the Blaine method and the data are shown in Fig. 4.

**Fig. 5:**
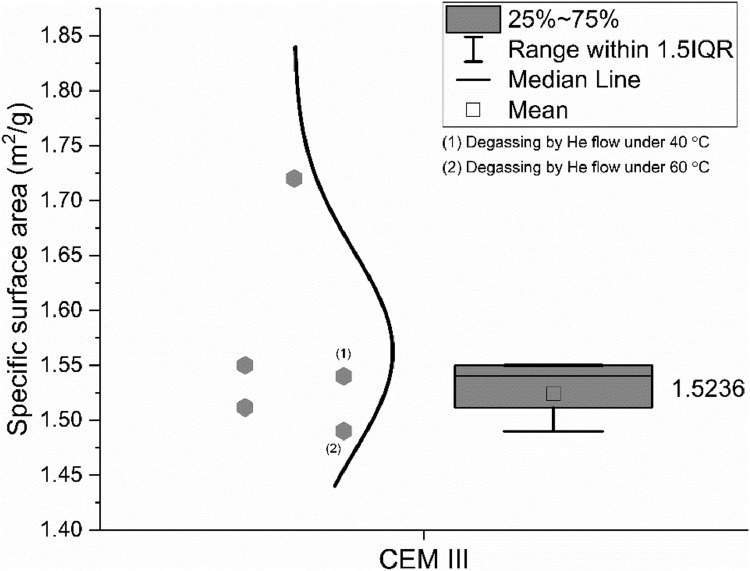
Specific surface area of the CEM III/A 42.5 N measured by the BET method.

The specific surface area of the CEM III/A 42.5 N was as well measured by the BET method, according to ISO 9277: 2010 [8]. Four different groups were involved in BET measurement and the data are shown in Fig. 5. The numbers in brackets indicate the values from the same sample but different pre-treatment methods that were conducted by the same group.

**Fig. 6:**
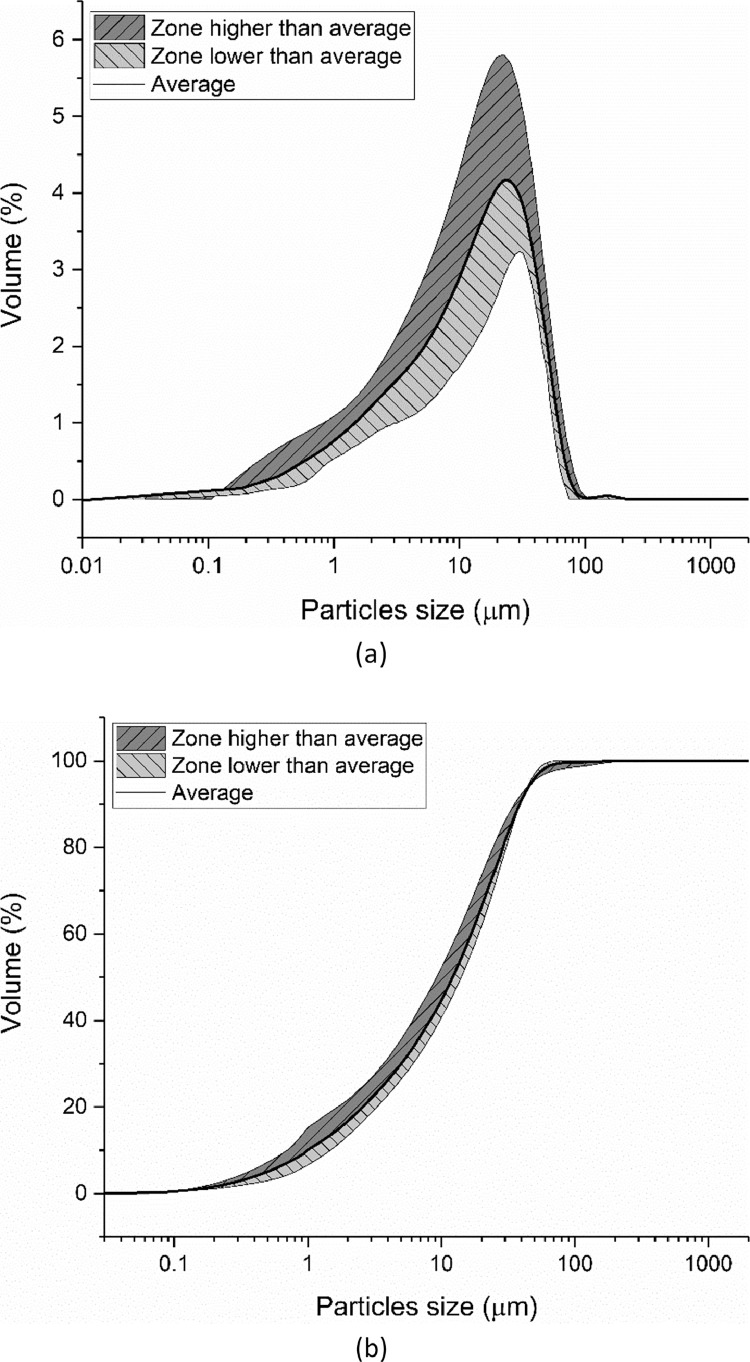
Particle size and distribution of CEM III/A 42.5 N measured by laser diffraction method; (a) Differential curve; (b) Integration curve.

**Fig. 7:**
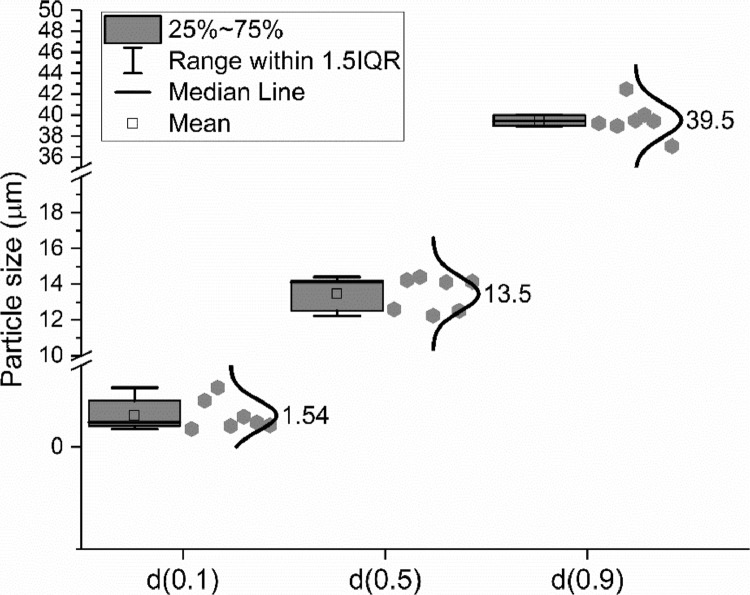
Particle sizes of the CEM III/A 42.5 N at d(0.1), d(0.5) and d(0.9).

Laser diffraction was applied to measure the particle size distribution (PSD) of the cement by eight different groups according to the method described in ISO 13320: 2009 [9]. Seven different groups conducted the particle size measurement and then the average distribution line was calculated, as shown in Fig. 6. The shadow areas below and above this average line indicate the scope of the testing results. The characterized particle size distributions (d(0.1), d(0.5) and d(0.9)) are shown in Fig. 7.

#### 1.1.3 Characterization data of other properties

**Fig. 8:**
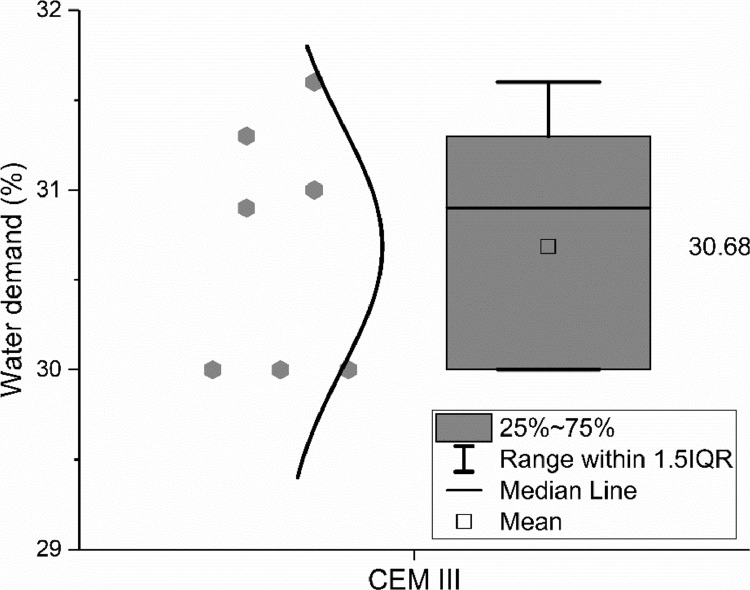
Water demand of the CEM III/A 42.5 N.

**Fig. 9:**
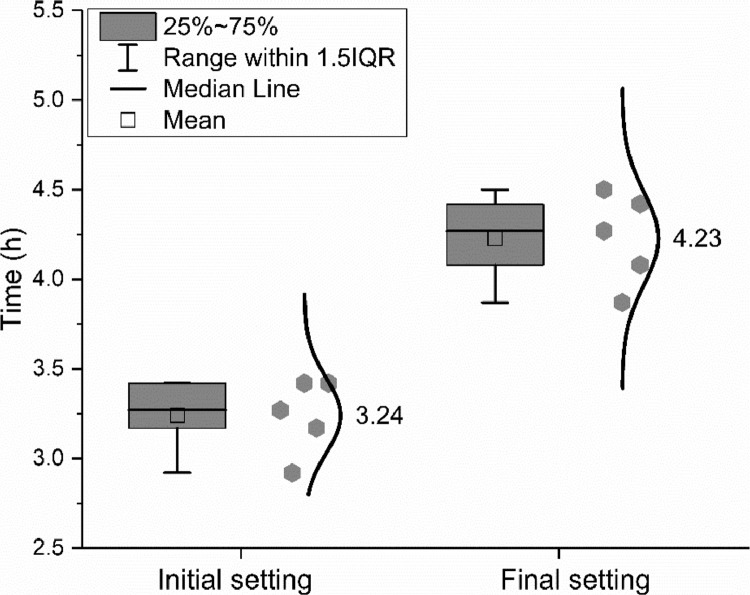
Initial and final setting time of the CEM III/A 42.5 N.

**Fig. 10:**
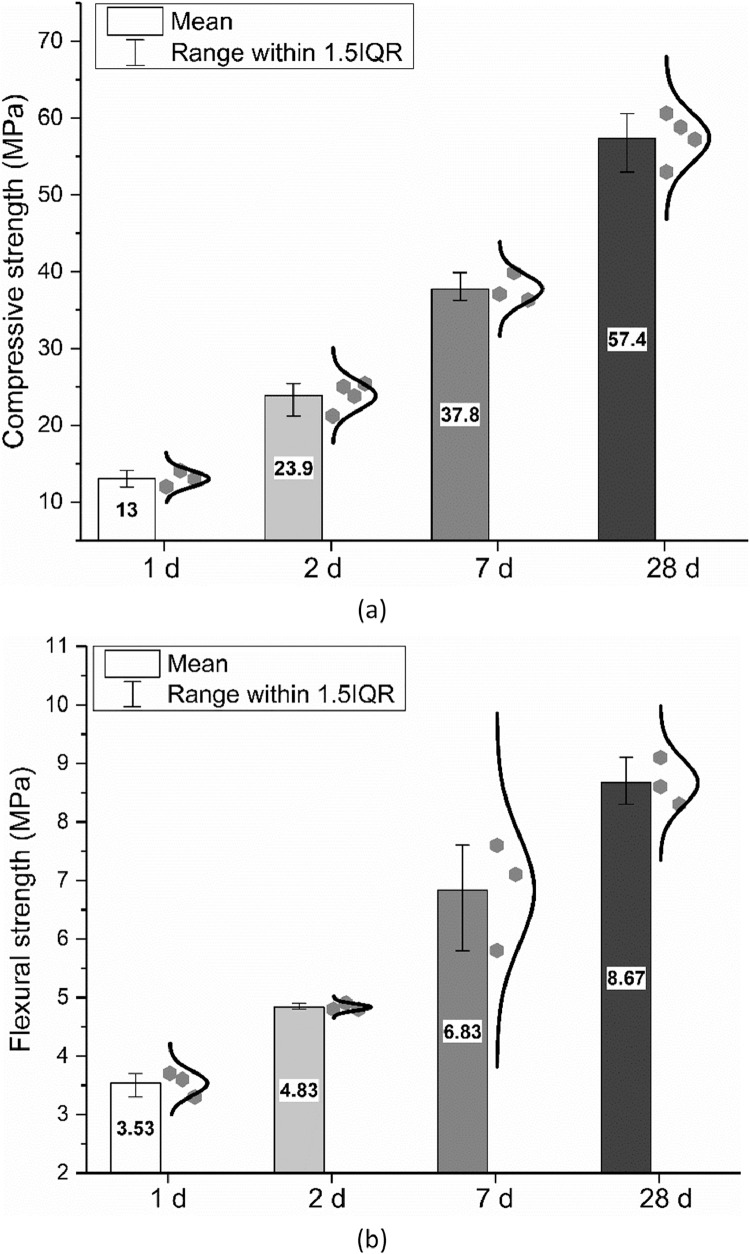
Mechanical strength of hardened cement mortars after curing for a certain time; (a) Compressive strength; (b) Flexural strength.

Water demand, initial and final setting time, were measured by seven and five different groups respectively according to EN 196–3: 2016 [10]. Flexural and compressive strength were measured by up to four different groups according to EN 196–1: 2016 [11]. The data are shown in Fig. 8, Fig. 9, Fig. 10.

**Fig. 11:**
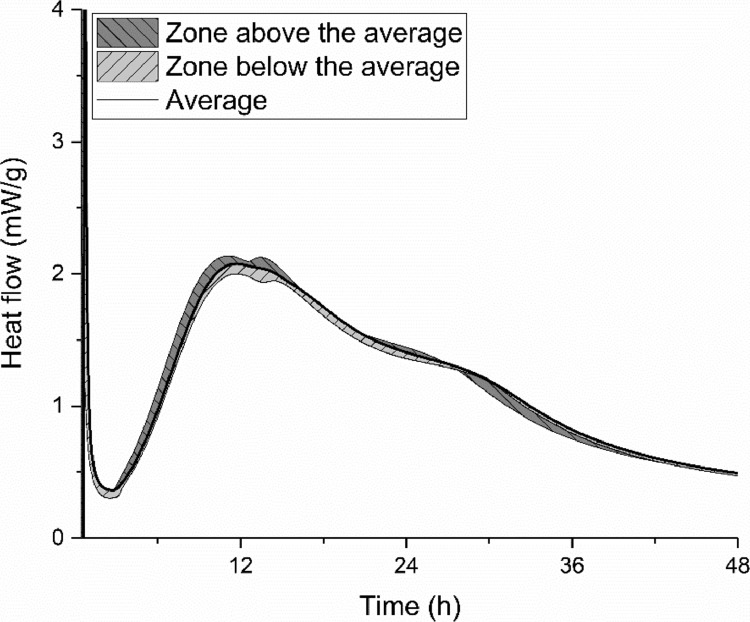
Isothermal calorimetry curve of cement paste with water to cement ratio of 0.434 at the temperature of 20 °C.

The cement hydration with a water to cement ratio of 0.434 at the temperature of 20 °C was monitored by three different groups according to EN 196–11: 2018 [12]. The data are shown in Fig. 11. The shadow areas below and above the average line indicate the scope of the test results.

### 1.2 Characterization data of slag

Slag is the second main constituent of CEM III. Hence the characterization of slag was conducted independently. Table 3 shows the data of the two groups who measured the composition by XRF and the LOI of slag. The phase contents and the selected physical properties of the slag are shown in Tables 4 and 5, which were contributed by one single group. In Group #2, the phase contents were determined by combining Rietveld refinement [3] with the external G-factor method [4] and are therefor absolute values.

**Table 3:** Oxide composition and LOI of slag measured by two different groups.

	CaO	SiO_2_	Al_2_O_3_	Fe_2_O_3_	MgO	K_2_O	Na_2_O	TiO_2_	P_2_O_5_	Mn_2_O_3_	SO_3_	LOI	Sum
#1	42.1	35.4	11.6	0.5	5.9	0.6	0.3	1.0	0.1	0.3	<0.2	0.57	98.70
#2	42.4	35.25	11.06	0.63	6.1	0.47	0.13	1.1	0.02	0.27	1.96	0.38	99.39

**Table 4:** Phase contents of slag measured by XRD.

	Calcite	Quartz	Alite	Belite	Slag	Sum
Content	3.3	0.9	0.4	0.1	95.3	99.9

**Table 5:** Physical properties of slag.

Slag	Density (kg/m^3^)	Specific surface area	
		Blaine method (cm^2^/g)	BET method (m^2^/g)
Physical properties	2.87	4684	2.38

**Fig. 12:**
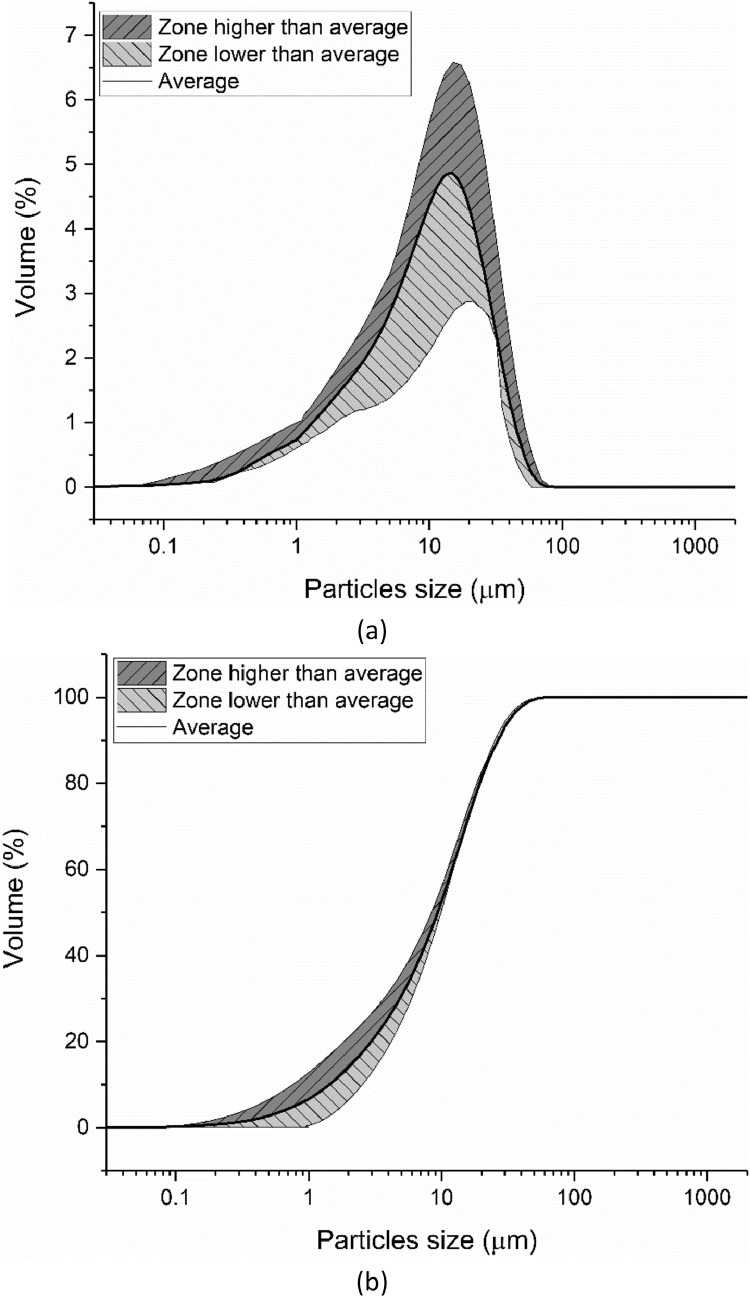
Particle size and distribution of the slag measured by laser diffraction method; (a) Differential curve; (b) Integration curve.

**Fig. 13:**
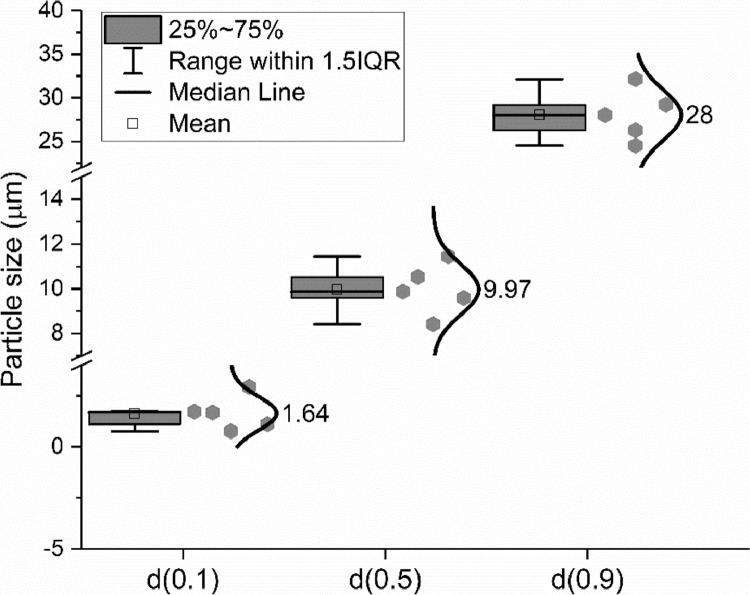
Particle size distribution of slag at d(0.1), d(0.5) and d(0.9).

The particle size distribution (PSD) of the ground slag was measured by the laser diffraction method. The data are shown in Fig. 12. The characterized particle size distribution of the slag (d(0.1), d(0.5) and d(0.9)) is shown in Fig. 13.

## 2 Experimental design, materials, and methods

For the characterizations of the CEM III/A 42.5 N, EN 196–2: 2013 was applied for the assessment of the oxide composition, insoluble residue, and loss on ignition. Density was measured according to EN 1097–7: 2008; specific surface area by the Blaine method was measured according to EN 196–6: 2018 and by the BET based on ISO 9277: 2010. Water demand and setting times were tested based on EN 196–3: 2016; flexural and compressive strength were obtained following EN 196–1: 2016. Isothermal heat flow calorimetry was measured according to EN 196–11: 2018. Particle size distributions were evaluated based on ISO 13320: 2009. For the other characterization methods of the CEM III/A 42.5 N, the specific experiment design and methods can be found in [1].

## Figures and Tables

**Table A.5:** Oxide composition of CEM III/A 42.5 N and the corresponding standard deviation.

	CaO	SiO_2_	Al_2_O_3_	Fe_2_O_3_	MgO	K_2_O	Na_2_O	TiO_2_	P_2_O_5_	Mn_2_O_3_	SO_3_*	SO_3_#	LOI	Cl^−^	Insoluble residue	Sum
Composition (wt.-%)	55.252	25.966	7.586	1.806	3.108	0.818	0.267	0.528	0.110	0.112	2.723	2.875	1.428	0.079	0.963	99.476
Standard deviation	0.663	0.131	0.216	0.162	0.098	0.075	0.014	0.035	0.052	0.010	0.005	0.130	0.504	0.003	0.104	0.329

⁎ Measured by XRF.

# Analysis by other methods.

**Table A.6:** Physical properties of CEM III/A 42.5 N and the corresponding standard deviation.

	Density (kg/ dm^3^)	Specific surface area* (cm^2^/g)	Specific surface area# (m^2^/g)	Particle size (μm)		
				d(0.1)	d(0.5)	d(0.9)
Average value	3.015	4282	1.523	1.54	13.5	39.5
Standard deviation	0.004	115.1	0.082	0.700	0.882	1.491

⁎ Measured by Blaine method.

# Measured by BET method.

**Table A.7:** Other properties of CEM III/A 42.5 N and the corresponding standard deviation.

	Water demand (wt.-%)	Setting time (h)		Compresive strength (MPa)				Flexural strength (MPa)			
		Initial	Final	1 d	2 d	7 d	28 d	1 d	2 d	7 d	28 d
Average value	30.68	3.24	4.23	13.03	23.85	37.77	57.40	3.53	4.83	6.83	8.67
Standard deviation	0.628	0.19	0.23	0.86	1.64	1.54	2.81	0.17	0.05	0.76	0.33

## Appendix

Average values and the standard deviation calculated based on the data from different groups (Tables A.5–Table A.6, Table A.7).

## Appendix B Supplementary material

Image, application 1

Image, application 2
